# Guided positional stabilization: a hypothesis on the role of targeted interfaces in enhancing upright stability among frail older adults

**DOI:** 10.3389/fragi.2026.1886928

**Published:** 2026-08-03

**Authors:** Mayur Bhatt, Harikrashna Bhatt

**Affiliations:** 1 Insightful Awareness, Sharon, MA, United States; 2 Brown University, Providence, RI, United States

**Keywords:** aging, fall prevention, frailty, light touch, postural stability, proprioception

## Abstract

Elderly adults are at a higher risk of falls due to many reasons including issues in balance, coordination and overall sensory responsiveness. Falls ultimately threaten health and result in the fear of falling this causes restricted mobility and further decrease in health. Although there are traditional interventions such as railings, walkers or balance therapy which aim to reduce falls, they often function on compensatory mechanisms with little for postural regulation. We hypothesize that Guided Positional Stabilization (GZ) which use light strategically situated contact interfaces, can improve upright stability in frail elderly individuals by stimulating proprioceptive and tactile pathways. GZ is not a support aid rather a neurosensory framework that engages the nervous system in reactive and anticipatory balance corrections. Learning from concepts in biomechanics, neuroscience and geriatrics, this article presents the design requirements, theory, and possible clinical applications of GZ in fall prevention. We also put forward a pilot research agenda for testing the effectiveness of GZ in real world environments.

## Introduction

Falls among the elderly population is a major concern with long term ramifications. Notably, 25% of adults over the age of 65 have encountered a fall and they are major cause of injury (Kakara et al., 2023). Along with resultant trauma, falls lead to reduced independence and poor quality of life (Burns et al., 2016). Subsequently as healthcare overall faces this shift to an elderly population, there is a pressing need for innovation and strategies to promote fall prevention.

Typical interventions include at-home modifications to assist in mobility. Furthermore physical therapy helps with balance, flexibility and strength. All of these modalities are not enough to address the ever changing and unpredictable reality of frailty and posture in an aging population (Gillespie et al., 2009). Specifically present day methods for fall prevention do little to utilize or perhaps reduce the individual’s neuromotor capabilities by swapping external support for internal regulation (Bateni and Maki, 2005).

Guided Positional Stabilization (GZ) offers a novel approach, rather than replacing internal mechanisms, it stimulates them. The underlying concept of GZ is the use of specific external interfaces to stimulate internal sensory feedback systems. There is intermittent and brief light contact that provides continuous stimulation of proprioceptive intake, allowing individuals to adjust their body posture and respond to any postural compromise. Notably, GZ excludes the requirement for full weight assistance and essentially maintains independence and encourages physical activity.

The primary innovation of Guided Positional Stabilization (GZ) does not lie in the invention of a new assistive device or sensing technology, rather in the conceptual integration of established neurophysiological principles into an environmental framework. Previous studies have demonstrated that light fingertip contact can reduce postural sway under controlled laboratory conditions. GZ extends these findings by proposing that properly positioned environmental interfaces can deliver intermittent tactile cues during routine daily movement, thereby supporting continuous neurosensory engagement throughout the built environment. Unlike traditional mobility aids, which primarily provide mechanical support, or isolated tactile cueing systems designed for specific neurological disorders, GZ emphasizes environmental design as a means of augmenting endogenous balance control while preserving user independence. This systems-level framework represents the main contribution of the proposed hypothesis.

### Definition of key terms

For the purposes of this hypothesis, targeted interfaces refer to intentionally positioned environmental structures designed to provide brief, light tactile contact during natural movement without functioning as weight-bearing supports. These interfaces may include vertically oriented poles or similar fixed architectural elements strategically located at transition points where postural adjustments frequently occur.

Light contact refers to minimal fingertip or hand contact that provides sensory input without meaningful mechanical support or transfer of body weight. The primary function of this contact is to enhance tactile and proprioceptive feedback rather than stabilize the body through external loading.

Frail older adults are defined as older individuals who exhibit age-related reductions in physiological capacity and increased functional decline, including impairments in balance, mobility, muscle strength, or sensorimotor function that increase susceptibility to falls.

Upright stability refers to the ability to maintain or recover stable standing posture during static or dynamic activities through effective sensorimotor integration and timely postural adjustments while minimizing body sway.

## Neurophysiological foundations of GZ in context of published data

Findings in human postural control have suggested the key role of somatosensory feedback in balance equilibrium. Receptors in the skin, are sensitive to subtle changes and can relay to the nervous system of changes in body position. Trials conducted in controlled settings suggest that even minimal fingertip contact with an immobile surface can significantly reduce motion during standing (Jeka and James, 1994). This is termed as ‘light touch stabilization’, emphasizes the importance of tactile cues to stabilize postural alignment without any mechanical support.

The proposed mechanism underlying GZ is based on the hypothesis that intermittent light contact with strategically positioned environmental interfaces increases the availability of somatosensory information during standing and walking. Initial contact activates cutaneous mechanoreceptors within the fingertips and palm, while simultaneously providing a stable spatial reference that supplements proprioceptive signals originating from muscle spindles, Golgi tendon organs, and joint receptors. These afferent inputs are integrated within spinal and supraspinal sensorimotor networks, including the cerebellum and somatosensory cortex, where they contribute to body orientation relative to the environment. Enhanced sensory integration is hypothesized to improve both anticipated and reactive postural adjustments by allowing earlier detection of fine deviations in body position. As a result, corrective muscle activation may occur before larger balance disturbances develop, thereby reducing postural sway and improving upright stability. This proposed pathway represents a hypothesis that warrants future study.

From a neurological point of view, the cerebellum and the somatosensory cortex receives input from the periphery such as Golgi tendon organs, joint receptors, and muscle spindles, which assist in the body’s spatial orientation. Proprioception allows for necessary adjustments in joint stiffness and muscle tone (Proske and Gandevia, 2012). Within the proposed GZ framework, intermittent tactile input is hypothesized to supplement these intrinsic proprioceptive signals by providing an external spatial reference. Rather than replacing endogenous balance mechanisms, this additional sensory information may reduce uncertainty in body-position, enabling the central nervous system to generate more accurate and timely posture corrections. In elderly individuals, proprioception is not as responsive due to decline in both central processing and peripheral nerves (Goble et al., 2009). Furthermore in one study, age-related deficits in muscle activation perception possibly contributed to declining neuromuscular control, postural instability, and increased fall risk in the elderly (Oleksy et al., 2025). GZ can respond to this decline by giving repeated sensory cues that reinstate the integrity of body posture.

Based on recent research in neurological rehabilitation, there is a suggestion that aged brains retain a certain amount of plasticity, especially under the exposure of repeated sensory stimuli. Tactile input can improve motor learning, integrate multiple stimuli and promote corticospinal excitability (Legon, 2009). These findings underscore the hypothesis that GZ when used consistently, can help increase stability not only during use but also in function when not being used.

## Design principles and configurations of GZ

Effective GZ implementation relies on several design principles intended to maximize reliable tactile feedback while reducing dependence on mechanical support. The interface should be positioned within comfortable reach during normal standing and ambulation so that users can establish brief contact without altering their natural gait. A vertical orientation is proposed because it accommodates contact across a range of body positions and movement trajectories while encouraging upright posture, in contrast to horizontal grab bars that are primarily intended for weight-bearing support. The recommended contact surface width of approximately 2–3 inches is intended as a preliminary design guideline rather than a fixed specification. This dimension is hypothesized to provide sufficient surface area for consistent tactile engagement while remaining narrow enough to discourage grasping or sustained weight-bearing. Future studies should determine the optimal dimensions for different patient populations.

The optimal installation height has not yet been established experimentally and should therefore be individualized according to user requirements. In general, interfaces should be positioned to facilitate upright standing posture. Future human-factors studies should establish standardized height recommendations based on user stature and functional capacity.

Second, the interface must provide the needed feedback. Materials such as carbon-fiber tubing, silicone composites or metallic poles with fine grooved patterns may significantly improve tactile perception. The interface should remain stable even under transient force application, as instability can reduce the reliability of sensory feedback. A mechanically stable interface is considered essential because inconsistent tactile input may reduce the reliability of afferent sensory signals required for accurate sensorimotor integration.

Third, GZ interfaces must be thoughtfully placed. Appropriate locations include areas between rooms, hallway corners, bathroom entries, and along routes to high-use zones such as kitchens and bedrooms. These areas often represent transition points where balance is most at risk due to changes in surface, lighting, or movement pattern (van Hoof et al., 2010). One study suggests that fingertip light touch with a fixed surface can improve sensory feedback, expand the limit of stability, and reduce postural sway, supporting its potential use as a low-load balance training strategy for the elderly (Tomita et al., 2024). This study underscores that strategically placed minimal-contact interfaces may enhance somatosensory feedback and improve balance control in older adults.

Finally, GZ must be person-centric. Devices should be adaptable to account for mobility degree, grip strength, and user height. Integration with smart sensors to track frequency and pattern of use may further personalize feedback and allow remote monitoring by caregivers.

## Clinical implications and use cases

GZ’s promise extends across diverse care settings. In long-term care facilities, where fall rates exceed those in community dwellings, GZ interfaces may serve as an intermediate layer of support between independence and full assistance. For example, installing vertical GZ poles near bed exits can reduce nocturnal falls, which are often precipitated by orthostatic hypotension or impaired spatial orientation.

In home environments, GZ may allow older adults to navigate familiar spaces while maintaining greater independence and minimizing reliance on conventional mobility aids. Strategically positioned tactile interfaces along commonly traveled routes, such as between the bedroom and bathroom or through hallways, are hypothesized to provide intermittent sensory cues that support postural orientation during everyday ambulation. Future prospective studies should evaluate whether such environmental modifications improve balance confidence, reduce postural sway, and decrease fall incidence in community-dwelling older adults.

GZ can also be used therapeutically. In outpatient rehab centers, therapists can use GZ interfaces during gait training to challenge dynamic balance while providing safety cues. GZ can complement dual-task training, where patients must simultaneously walk and perform cognitive tasks—a known stressor for postural control in frail adults (Beauchet et al., 2005).

For individuals with dementia, GZ may provide more than balance support. Repeated contact with familiar textures and structures can act as procedural memory triggers, enhancing orientation and reducing agitation. Color-coded GZ poles may further aid recognition and compliance among those with moderate cognitive impairment (Lord et al., 1994).

## Comparison with existing approaches

When contrasted with traditional fall prevention tools, GZ occupies a unique niche. Unlike canes or walkers, it does not substitute for postural effort but augments it. Unlike grab bars, it is designed for transient interaction, minimizing the risk of learned helplessness or overdependence. Unlike wearable sensors, GZ does not require technological literacy or maintenance, making it particularly suitable for older adults who may resist or forget to use active systems.

Quantitative comparison may rely on variables such as reduction in sway area (measured via force plates), improved Timed Up and Go scores, or decreased frequency of minor stumbles. GZ’s efficacy may also manifest through qualitative indicators such as increased gait confidence, reduced caregiver supervision time, or enhanced participation in daily activities.

While GZ shares some principles with tactile cueing devices used in Parkinson’s disease (e.g., laser canes), it differs in being environment-embedded rather than user-worn. This distinction is critical, as it reduces the cognitive load on the user and lowers device rejection rates (Maki and McIlroy, 2006).

## Discussion and proposed future directions

The introduction of GZ into the field of fall prevention invites a paradigm shift. Rather than treating balance loss as an inevitable consequence of aging, GZ reframes stability as a trainable, improvable function even in the context of frailty. By using the environment as a partner in neurosensory support, GZ upholds principles of aging-in-place, autonomy, and human dignity.

A logical next step is a prospective pilot study designed to evaluate the feasibility, safety, and preliminary efficacy of GZ in community-dwelling and assisted-living older adults. Eligible participants could include adults aged 65 years or older who demonstrate impaired balance, mild-to-moderate frailty, or an elevated risk of falls while remaining capable of independent or minimally assisted ambulation. Individuals with severe cognitive impairment, profound peripheral neuropathy, complete dependence for ambulation, or unstable medical conditions that substantially affect balance would be excluded. Participants could be randomly assigned to either a GZ-modified environment or a control environment receiving standard environmental modifications without strategically positioned tactile interfaces.

Primary outcome measures could include changes in postural sway, Timed Up and Go performance, fall incidence, and fear of falling as assessed using validated instruments such as the Falls Efficacy Scale. Secondary outcomes may include gait confidence, adherence to GZ interface use, quality of life, functional mobility, and user satisfaction. Quantitative measures of interface utilization obtained through optional sensor integration may also provide insight into adherence and interaction patterns.

Integration into existing infrastructure poses both challenges and opportunities. Healthcare facilities may need to revise building codes or aesthetic standards, while manufacturers must develop universal mounting systems. Insurance coverage and Medicare reimbursement will hinge on demonstrated cost-effectiveness, which can be estimated through health economic modeling based on reduced injury incidence.

GZ is not a panacea. In individuals with profound neuropathy, vestibular deficits, or severe dementia, the efficacy of tactile cueing may be limited. Moreover, improper installation or maintenance may lead to unintended hazards. Therefore, protocols must be developed for safe deployment, user training, and feedback loops to monitor ongoing performance.

## Conclusion

Guided Positional Stabilization (GZ) introduces a compelling hypothesis: that strategic, minimal-contact interfaces can enhance upright balance and reduce fall risk in frail older adults by activating dormant or underutilized sensorimotor pathways. Rooted in neuroscience and aligned with human-centered design, GZ offers a middle path between unsupported ambulation and full physical assistance. Its applications span home, clinical, and rehabilitative settings. Through structured research, interdisciplinary collaboration, and iterative refinement, GZ may evolve from theoretical construct to practical intervention with global impact.

## Data Availability

The original contributions presented in the study are included in the article/supplementary material, further inquiries can be directed to the corresponding author.

## References

[B1] Bateni H. Maki B. E. (2005). Assistive devices for balance and mobility: benefits, demands, and adverse consequences. Archives Physical Medicine Rehabilitation 86 (1), 134–145. 10.1016/j.apmr.2004.04.023 15641004

[B2] Beauchet O. Dubost V. Herrmann F. R. Kressig R. W. (2005). Stride-to-stride variability while backward counting among healthy young adults. J. Neuroengineering Rehabilitation 2 (1), 26. 10.1186/1743-0003-2-26 16095533 PMC1190208

[B3] Burns E. R. Stevens J. A. Lee R. (2016). The direct costs of fatal and non-fatal falls among older adults—United states. J. Safety Research 58, 99–103. 10.1016/j.jsr.2016.05.001 27620939 PMC6823838

[B4] Gillespie L. D. Robertson M. C. Gillespie W. J. Sherrington C. Gates S. Clemson L. (2009). Interventions for preventing falls in older people living in the community. Cochrane Database Systematic Reviews 2. 10.1002/14651858.CD007146.pub3 22972103 PMC8095069

[B5] Goble D. J. Coxon J. P. Wenderoth N. Van Impe A. Swinnen S. P. (2009). Proprioceptive sensibility in the elderly: degeneration, functional consequences and plastic-adaptive processes. Neurosci. & Biobehav. Rev. 33 (3), 271–278. 10.1016/j.neubiorev.2008.08.012 18793668

[B6] Jeka J. J. James R. L. (1994). Fingertip contact influences human postural control. Exp. Brain Research 79 (2), 495–502. 10.1007/BF02738408 7813685

[B7] Kakara R. Bergen G. Burns E. Stevens M. (2023). Nonfatal and fatal falls among adults aged≥ 65 years—United states, 2020–2021. MMWR. Morb. Mortality Weekly Report 72, 938–943. 10.15585/mmwr.mm7235a1 37651272

[B8] Legon W. (2009). Sensory Information to Motor Cortices: Effects of Motor Execution in the upper-limb Contralateral to Sensory Input.

[B9] Lord S. R. Ward J. A. Williams P. Anstey K. J. (1994). Physiological factors associated with falls in older community‐dwelling women. J. Am. Geriatrics Soc. 42 (10), 1110–1117. 10.1111/j.1532-5415.1994.tb06218.x 7930338

[B10] Maki B. E. McIlroy W. E. (2006). Control of rapid limb movements for balance recovery: age-related changes and implications for fall prevention. Age Ageing 35 (Suppl. 2), ii12–ii18. 10.1093/ageing/afl078 16926197

[B11] Oleksy Ł. Mika A. Sopa M. Stolarczyk A. Adamska O. Zyznawska J. (2025). Proprioceptive control of muscle activation in aging: implications for balance and fall risk. Biology 14.6, 703. 10.3390/biology14060703 40563954 PMC12189530

[B12] Proske U. Gandevia S. C. (2012). The proprioceptive senses: their roles in signaling body shape, body position and movement, and muscle force. Physiol. Reviews 92 (4), 1651–1697. 10.1152/physrev.00048.2011 23073629

[B13] Tomita H. Asai H. Ogawa Y. Kawamata N. Hayashi H. (2024). Fingertip light touch contact increases anteroposterior limits of stability in healthy young and older adults. Gait & Posture 114, 28–34. 10.1016/j.gaitpost.2024.08.081 39217814

[B14] van Hoof J. Kort H. S. van Waarde H. Blom M. M. (2010). Environmental interventions and the design of homes for older adults with dementia: an overview. Am. J. Alzheimers Dis. Other Demen 25 (3), 202–232. 10.1177/1533317509358885 20150655 PMC10845627

